# No apparent transmission of livestock-associated methicillin-resistant *Staphylococcus aureus* CC398 in a survey of staff at a regional Danish hospital

**DOI:** 10.1186/s13756-017-0284-y

**Published:** 2017-12-13

**Authors:** Else Toft Würtz, Jakob Hjort Bønløkke, Tinna Ravnholt Urth, Jesper Larsen, Md Zohorul Islam, Torben Sigsgaard, Vivi Schlünssen, Troels Skou, Anne Mette Madsen, Louise Feld, Saloomeh Moslehi-Jenabian, Robert Leo Skov, Øyvind Omland

**Affiliations:** 1 Department of Occupational Medicine, Danish Ramazzini Centre, Aalborg University Hospital, Havrevangen 1,4, 9000 Aalborg, Denmark; 2 Section of Environment, Work and Health, Department of Public Health, Danish Ramazzini Centre, Aarhus University, Aarhus, Denmark; 3 Microbiology and Infection Control, Statens Serum Institut, Copenhagen, Denmark; 4 The National Research Centre for the Working Environment, Copenhagen Ø, Denmark; 5 Department of Clinical Medicine, Aalborg University, Aalborg, Denmark

**Keywords:** Animal farming, Bacterial transmission, Dust, Hospital employees, Hospital environment, LA-MRSA, Nasal swabs

## Abstract

**Background:**

In recent years, livestock-associated methicillin-resistant *Staphylococcus aureus* (LA-MRSA) multi locus sequence type CC398 has spread widely in the livestock production in Europe. The rates of LA-MRSA in hospitals have been found to be largely determined by contact to and density of livestock in the area.

**Methods:**

This is a cross sectional study of the prevalence of LA-MRSA among hospital staff in a Danish hospital situated in a livestock production region. We analysed nasal swabs, air and dust samples for the presence of MRSA using PCR and mass spectrometry.

**Results:**

Of 1745 employees, 545 (31%) contributed nasal swabs. MRSA was not detected in any participant, nor was it detected in air or dust at the hospital or in houses of employees living on farms. Four percent of the participants had contact to pigs either directly or through household members. LA-MRSA was detected in two of 26 samples from animal sheds, both of them from pig farms. The participation rate was relatively low, but participants were representative for the source population with regards to animal contact and job titles.

**Conclusions:**

The study suggests a low point prevalence of LA-MRSA carriage in Danish hospital staff even in regions where livestock production is dense. Should more studies confirm our findings we see no need for additional hospital precautions towards LA-MRSA in Denmark at the moment. We think that our data might reduce potential stigmatization of hospital workers with contact to LA-MRSA positive farms at their work places and in their communities.

**Electronic supplementary material:**

The online version of this article (10.1186/s13756-017-0284-y) contains supplementary material, which is available to authorized users.

## Background

The predominant livestock-associated methicillin-resistant *Staphylococcus aureus* (LA-MRSA) in Europe are strains of clonal complex 398 (CC398) [1, 2]. In European pig production LA-MRSA essentially equals CC398 whereas in North America there is greater variation in the LA-MRSA strains [3] and in Asia the LA-MRSA strains ST9 and ST221 dominate [4]. A Dutch study showed that the distribution of human LA-MRSA isolates corresponded to the density of livestock farming (pig and cattle) while the distribution of non-zoonotic MRSA cases corresponded to the density of the human population [5]. Transmission of LA-MRSA CC398 between humans appears to occur less easily compared with human MRSA strains [6–11]. In Denmark, the colonization and infection rates with LA-MRSA CC398 in humans have steadily increased [12, 13] though recently the increase in the incidence of LA-MRSA CC398 infections seems to level of [13]. The association between frequent exposure to LA-MRSA and persistent colonization is well established in farmers, but a distinction between repeated contaminations and persistent colonization is complicated [2, 14]. Nevertheless, in a study of short-term occupational exposure to livestock the presence of LA-MRSA was rarely observed for more than 24 h [14]. Thus, LA-MRSA carriage among household members of farmers may depend strongly on repeated animal exposure in the farmer [15]. A Danish study revealed a clear temporal and spatial association between LA-MRSA infections among subjects with direct or indirect livestock contact and subjects with no livestock exposure [12]. The pattern of transmission observed in Denmark resembles that seen in other countries with intensive livestock production [2]. Since the 1980s and with continuous updates, the Netherlands and Denmark have implemented so-called ‘search and destroy’ policies, to prevent transmission of any MRSA into hospitals and other healthcare facilities [16]. This strategy has been highly successful with very low incidences of MRSA infections in Danish and Dutch hospitals [16]. However, introduction of LA-MRSA into hospitals by staff with direct or indirect livestock contact has become a potentially important route of transmission. A healthcare worker was thus assumed to be the source related to the first LA-MRSA outbreak in a Dutch hospital [17]. In a previous study, the incidence of LA-MRSA carriage in Dutch healthcare workers with direct or indirect contact to pig or veal calves was shown to be low (1.7%). Nevertheless, MRSA carriage was 10-fold higher than in healthcare workers without livestock contact (0.15%) [18]. In light of the Dutch observations, that transmission of LA-MRSA by healthcare workers with livestock contact could not be excluded, we wanted to measure the point prevalence of LA-MRSA among hospital staff members in a Danish rural region with high pig density. Furthermore, we wanted to analyse for the effect of the Danish “search and destroy” policy by measuring any MRSA in dust samples in the hospital environment.

## Methods

### Setting

The study was conducted at Hjørring Hospital, which at the time was part of Vendsyssel Hospital with a catchment population of approximately 200,000 inhabitants of the North Denmark Region. The North Denmark Region covers 18% of the Danish area, includes 10% of the Danish population, and 23% of farms with livestock (20% of all pig farms) numbers available on Statistics Denmark [19] corresponding to an area of high agricultural density. All 1745 employees (including approx. 240 students), were invited to participate in the study that took place over a 3-week period in the autumn of 2015 during normal working hours between 7 am and 8 pm. Information about the study was presented at the hospital’s intranet in text, by a short movie, and through announced meetings. The consecutive inclusion of participants extended for three weeks in September 2015.

### Study design

This cross-sectional study was composed of three major parts: i) A questionnaire directed to the employees and a nasal swab analysis; ii) Environmental dust sampling at the hospital; and iii) Dust sampling in the household of the participants (bedrooms) and if relevant related animal sheds.

### Questionnaire and nasal swabs

All participants received the questionnaire by e-mail for electronic completion or as handouts. The questionnaire included items of e.g. sex, age, job category, direct or indirect contact (through household members) with animal production (primarily pigs, cattle, poultry and mink) and companion animals, self-evaluated health, earlier disease with a focus on skin disease, and ever having been carrying or infected with MRSA CC398.

Nasal swabs were taken from the anterior nares by the investigators using the ESwab liquid-based collection and transport system (Copan Innovation, Italy) according to the manufacturer’s instructions. Nasal swabs were stored in transport medium (Amies) and processed within 3–4 days at Statens Serum Institut (SSI), Copenhagen, Denmark.

Data were obtained from Statistics Denmark on the proportion of households with members employed in livestock farming in the region of Denmark where staff from the hospital lived.

### Environmental dust sampling at the hospital

Short-term sampling of bacteria in bioaerosols was carried out on September 14th and 15th 2015 at the hospital. Four types of active samplers were used (details described later). A total of 20 different areas at the hospital and two reference areas outside the hospital were selected for sampling. Each type of active sampler was placed in at least five and at most 17 of the selected areas so that each area was sampled by at least one and at most five different methods. The presence of employees, patients and visitors in each sampling area during the sampling period was noted, and categorized into none, less than 5, 5 to 10, 10 to 20, and more than 20 persons. Average temperature and relative humidity was measured in the sampling locations during sampling.

Long-term sampling of bioaerosols was performed using passive sampling from September 14th to October 7rd 2015. Both active and passive samplers were placed 1.4 to 2.0 m above ground level. The positions included four locker rooms, two in the bed cleaning areas, nine in different waiting rooms or areas designated for patient reception, two areas in the basement where patients, beds and laundry were transported, one consulting room, one lunchroom, one lunch desk, one main entrance and the outdoor reference.

### Dust sampling in homes and related animal sheds

**Fig. 1:**
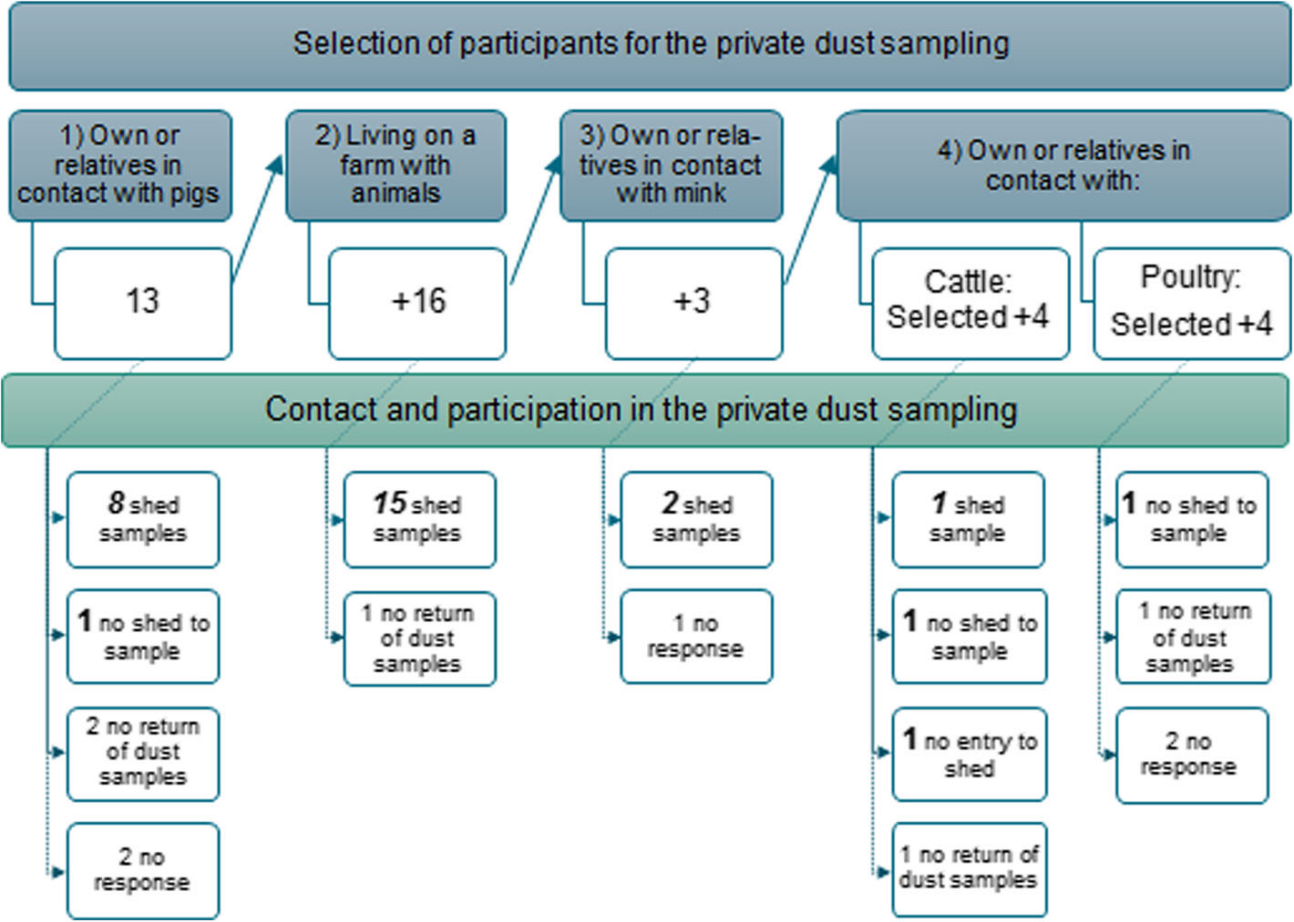
Selection of dust samplings and collected samples from private homes and sheds. 40 participants invited to the private dust sampling. Collected samples: home (bedroom) dust samples *n* = 30 (bold), shed dust samples *n* = 26 (bold and italics)

According to the study protocol, 40 participants were to be selected for the sampling of dust in their home and, if relevant in an animal shed: 20 LA-MRSA carriers and 20 non-carriers. Additionally, 10 participants in each group should be in direct or indirect contact with production animals and 10 should be without animal contact. As all the nasal swabs were negative for LA-MRSA (see below), the selection of participants for the home and farm dust sampling was changed, as illustrated in Fig. 1. The final groups were not as intended mutually exclusive, but were selected based on questionnaire information with the aim to include those participants with most contact with pigs, other farm animals or farmers, based on assumed highest LA-MRSA exposure. First, participants with direct or indirect contact with pigs were identified, *n* = 13. Secondly, participants living on a farm with animals (including hobby farms) were selected, *n* = 16. The third group of participants were those with direct or indirect contact with mink, *n* = 3. Finally, participants with direct or indirect contact with cattle, *n* = 4, and poultry, n = 4 were selected in order to obtain 40 locations for dust sampling. The home dust sample was collected from the bedroom. The animal shed samples were collected from buildings with animals, representing confinement buildings, stables, mink farms as well as small hobby farms. The dust samples from the bedrooms and the animal sheds were collected over 14 days during October–December 2015.

### Dust samplers and extraction methods

Four types of active samplers were used in parallel to collect airborne bacteria. Inhalable Gesamtstaubprobenahme samplers (GSP; BGI Inc., Waltham; MA, USA) and IOM (SKC Inc., PA, USA) samplers mounted with 1.0 μm pore size polycarbonate filters (Maine Manufacturing, Sanford, USA) were used at a flow rate of 3.5 l/min and 2.0 l/min, respectively. Sampling was performed in 17 areas with an average sampling period of 118 min. Bacteria from filters were extracted by orbital shaking in a pyrogen-free 0.05% Tween 80 and 0.85% NaCl solution as described previously [20]. The BioSampler (SKC Inc., PA, USA) was used for direct sampling in 20 ml pyrogen-free solution (0.001% Tween 80, 0.85% NaCl). The BioSampler was used for sampling in 5 areas at a flow rate of 7.5 l/min for an average of 50 min during which the sampler was cooled with cooling elements. The BioSampler samples were cultured within 2 h after sampling. All samples from the GSP, IOM, and BioSampler were cultured on Nutrient agar (Oxoid, Basingstoke, UK) supplemented with actidione (50 mg/l cycloheximide) at 25 °C for general cultivation of bacteria (NA plates), on SaSelect agar plates (Bio-Rad, France) at 37 °C for selection of staphylococci (SA plates) and on MRSA–selective agar plates (Oxoid, United Kingdom) at 37 °C for selection of MRSA (MRSA plates). Bacterial colonies were counted after 7 days for NA plates and after 24 h for SA and MRSA plates.

Airborne bacteria were also sampled using a Six-stage Viable Andersen Cascade Impactor (ACI) (N6, Thermo Fisher Scientic Inc. Waltham, MA, USA) in 11 of the areas at a flow rate of 28.3 l/min for 20 min on MRSA plates and for 5 min on NA plates. For calculation of number of colony forming units from the ACI samples, data for the six levels of the sampler were pooled.

EDC samplers (electrostatic dust collectors, ZEEMAN Alphen, the Netherlands) were used for long term sampling as described previously [21] in 8 areas of the hospital and for the dust sampling from bedrooms and animal sheds. EDCs from the hospital were extracted as described by Madsen et al. [22] and cultured on SA and MRSA plates as described above.

EDCs from the bedrooms and animal sheds were extracted as described by Shorter et al. [23]. In brief, the EDCs were extracted twice in 50 mL sterile water with 0.05% Tween20. The extracts were concentrated using centrifugation and subjected to beat milling for mechanical cell disruption before DNA purification. They were then processed similar to nasal swab samples as described in the next section.

### MRSA identification and characterization

MRSA was identified according to standard laboratory methods. Nasal swabs and dust samples from EDCs in homes and animal sheds were analysed at SSI. In brief, 200 μl of nasal swab transport medium or EDC extract was enriched in 5 ml of Mueller-Hinton broth (Sigma Aldrich) containing 6.5% NaCl for 18 h at 35 °C. Ten microliters of enriched broth were cultured on Brilliance MRSA 2 agar plates (Oxoid, United Kingdom) and incubated for 20 h at 35 °C. Presumptive MRSA colonies were streaked onto blood agar and grown overnight at 35 °C.

MRSA was identified and characterized using a multiplex PCR assay as described previously [24]. *spa* typing was performed using the Ridom Staph Type standard protocol [25] and the Ridom SpaServer [26].

### Identification of bacteria by MALDI-TOF mass spectrometry

Bacteria colonies from the indoor hospital environment were identified using Matrix-assisted laser desorption-ionization time-of-flight (MALDI-TOF) mass spectrometry (Bruker Daltonics, Bremen, Germany) using Bruker Biotyper 3.1 software with the BDAL standard library. Bacterial colonies from the agar plates were prepared using the extended direct transfer methods as described earlier [27].

### Statistics

A two-sample test of proportions was used to test if the distribution of job categories differed between the responders and the entire population of staff and between the individual job categories. The same test was used to validate the proportion with agricultural contact between responders and staff through data from Statistics Denmark. Descriptive statistics were used for the remaining results. The significance level was set at 5%. Statistical analyses were conducted in Stata 12.1 (StataCorp LP, 2011).

## Results

A total of 546 (31.3%) of the 1745 hospital employees agreed to participate. Of the 546 who agreed, one missed the swab collection and 36 did not return the questionnaire despite two reminders. Thus, we obtained information from a total of 509 employees. Among the participants the proportion of physicians was smaller compared to the proportion of physicians at Hjørring Hospital (7.6% vs 13.2% (*p* < 0.001)). The other groups of employees were distributed similar to their distribution in the total staff. The prevalence of staff having a household member working as farmer (6.7%) was as expected according to the estimated proportion from Statistics Denmark of hospital employees with a household member working in agriculture of 6.0% (*p* = 0.28).

In total 505 participants answered the residence item in the questionnaire of whom 26 (5.1%) lived on operative farms. Direct and/or indirect contact with pigs was reported by 21 (4.1%), while 28 (5.5%) reported contact to cattle, 7 (1.4%) to mink, 38 (7.5%) to poultry, and 67 (13.1%) to horses. Overall 111 (21.8%) had direct or indirect contact to either pigs, cattle, mink, poultry or horses as some participants reported contact with more than one type of animal.

All of the 545 collected swabs from the hospital staff were negative for any MRSA including LA-MRSA CC398.

### Environmental dust sampling at the hospital

MRSA including LA-MRSA was not found in the indoor air samples at the hospital at any time or place during the two-day sampling period with active bioaerosol samplers. Neither was MRSA/LA-MRSA found during the prolonged 23-day sampling period with the passive EDC samplers.

In contrast to *S. aureus*, other types of staphylococci were found in the examined hospital areas. In total nine different *Staphylococcus* species were found using different active samplers [see Additional file 1]. Using passive long-term samplers, five different *Staphylococcus* species were found [see Additional file 2].

The hospital indoor temperatures during sampling were on average 21.1 °C and 24.1 °C on the two sampling days and the relative humidity was on average 59% on both days.

### Dust sampling in homes

52% of the participants agreed to contribute to home dust sampling (65% of those living on operative farms and 51% of those not living on operative farms, *p* = 0.16). The collection of dust samples is included in Fig. 1. We were unable to contact five of these participants and five missed to return the EDC samplers. Three participants did not have contact to an animal shed and one animal shed was considered unavailable because of a diseased family member. In total 30 EDC samples from bedrooms and 26 animal shed EDC samples were obtained. All of the 30 EDCs from the homes of hospital staff members were negative for MRSA including LA-MRSA. The animal sheds covered both operative farms as well as hobby farms with small sheds housing animals for leisure activities. The 17 operative farms included in the dust sampling represented six pig farms, seven dairy/cattle farms and four mink farms. Two of the 26 samples from animal sheds and farms were positive for LA-MRSA CC398 with *spa*-type t034. Both of these were from pig farms where members of a participant’s household worked but where the participating hospital employee had no direct contact with the pigs.

## Discussion

Different approaches were used to identify LA-MRSA in this study: Nasal swabs from hospital staff members, samples from the indoor hospital environment, and samples from homes of selected hospital staff members and animal sheds related to the household of these staff members. We found LA-MRSA in two out of six pig sheds. We did not find any MRSA isolates in the 20 other animal sheds, among the hospital staff members, in the indoor hospital environment, or bedrooms of the hospital staff.

In contrast to recent studies [10, 28, 29] that have reported prevalence’s between 4 and 16% of LA-MRSA in household members of livestock farmers, we identified no LA-MRSA carriers among household members. Considering that only 26 participants reported living on operative livestock farms, even the highest reported 16% prevalence of LA-MRSA carriage would have meant that we only should have expected four cases in our study. Larger studies would be necessary in order to investigate the current LA-MRSA carriage rates with reasonable statistical power in the Danish population. A small pilot study revealed a lower MRSA carriage prevalence among pig farm household members in Denmark compared to Belgium and the Netherlands [15]. Others have also found a low carrier prevalence of LA-MRSA in healthcare workers associated to livestock farming households. In a Dutch study published in 2008 where 4.4% of 855 healthcare workers had direct or indirect contact with pigs or veal calves, only one person carried LA-MRSA as well as one person without any livestock contact [18]. We speculate therefore that the prevalence of LA-MRSA carriage in Danish healthcare workers with livestock farm contact may be low compared with other recent European studies and that our negative findings reflect a truly very low prevalence of LA-MRSA carriage among the hospital staff. The latter assumption is supported by the negative findings in the environmental samples taken from the hospital. Therefore, we believe that the lack of LA-MRSA in samples taken at the hospital is not due to limited contact of staff with farms. Rather that it reflects that LA-MRSA carriage from farms and farm animals is unusual among staff despite of having contact to animal or contact with family members working on farms.

The study was not designed to detect the prevalence of LA-MRSA in farms. The prevalence of 33% LA-MRSA positive swine farms that we observed is highly uncertain because only six farms were tested and because of possible skewed participation among farms. After initiation of the study, screenings showed that in Denmark in 2014, 68% of conventional pig herds, 6% of organic pig herds, 10% of veal calf herds, and 16% of mink herds were LA-MRSA positive [13].

For the hospital environmental samples no bacteria grew on the MRSA selective agar and no *S. aureus* was found on the selective agar. There is no gold standard method for sampling airborne MRSA so we decided to strengthen the study design by applying a number of different sampling methods and locations in the study: Active sampling on agar, filters and in an aqueous solution, and additional passive sampling on electrostatic cloths. As no MRSA was found, data for all active samplers were pooled. In total nine different *Staphylococcus* species were found. Different bacterial species were found especially in the lunchroom where many people were present. The species included different common human skin-related species such as *S. epidermidis*, but also soil related bacteria such as *Bacillus licheniformis*. As we were able to identify several staphylococcal and skin-related bacterial species, we assume that if LA-MRSA had been present in similar concentrations we would have identified it with the methods applied. We expect that if we had been taking swabs from surfaces such as, e.g. door handles the likelihood of detecting *S. aureus* would have been increased.

Recent studies in livestock dense regions in Germany and Spain found that although the majority of CC398 MRSA in hospitalized patients was found among subjects with farm or livestock contact [30] or that CC398 was the most prevalent MRSA strain among patients with livestock contact [31] smaller proportions of CC398 were found without indications of transfer from livestock. This is a possible indication of human-to-human transmission of the bacteria outside or even within the hospital environment. However, research suggests that LA-MRSA is less transmissible than other MRSA in hospitals [7]. As transmission rates do not necessarily remain stable over time there is a need for continuously studying transmission rates of different strains of MRSA in hospitals and to review preventive measures accordingly. Our study suggests that the preventive measures currently in place in the hospital we investigated do not need to be strengthened.

Strengths of the study is the size of the study, the 3-stage design, and the comprehensive measuring program that increased the possibility to track a potential route of LA-MRSA transmission from a local farm to the hospital if present. It is an additional strength that we were able to include employees in the study with a distribution of jobs almost similar to the source population at the hospital and that the prevalence of farmers was as expected according to data for the region from Statistics Denmark thus reducing the risk of bias due to skewed recruitment.

A clear limitation is the low participation rate of only 31% when both employees and students were considered. Ineffectual study information might be the reason for both the general low participation rate and the few LA-MRSA positive pig farms. The fear of stigmatization at the hospital and in the form of social isolation in the local community may have kept subjects from participating even though the information of carrier status and of LA-MRSA status in animal sheds, as clearly stated to the participants, was only available to the principal investigator and was quickly anonymized. If such a fear among the hospital staff was indeed present, it would have led to less participation among subjects living on farms known to host LA-MRSA. Anecdotally, the investigators heard stories from participants both of colleagues living on farms who did not want to participate and of colleagues from livestock farms who expressed strong obligations to participate because of their farm contact.

Another limitation is the fact that we did not perform swabs of the throat and perineum of the participants, nor from surfaces at the hospital. The sensitivity of nasal swabs alone is reported in the range of 66–93% [32–35], increasing to 95–98% when swabs are performed from throat and perineum as well [34, 36]. Given the low prevalence of MRSA in samples from animal sheds and the lack of MRSA in samples from homes and the hospital, we do not believe that using only one nasal sample per participant has had a major influence of the results. Furthermore, we only tested the nasal swabs for MRSA and not for *S. aureus* as such.

Sampling was not repeated but was only done once, and the result is thus a point estimate of the LA-MRSA carriage. However, we have no knowledge, as to what extent seasonal variation influence the human transmission. Had we found LA-MRSA in the nasal swabs it would have been possible to genotype the bacteria and follow its route of transmission from farm samples to hospital.

## Conclusions

Neither the hospital staff nor the hospital environment of the participating regional Danish hospital appeared to host LA-MRSA or other MRSA at the time of this investigation. We identified no transmission route of LA-MRSA from households or farms by staff members to the hospital. Based on this cross-sectional study from a single hospital the risk for LA-MRSA transmission from livestock farms via hospital staff to Danish hospitals is regarded as low, even in areas with LA-MRSA positive farms. However, the findings are based on a limited number of observations. Should more studies confirm our findings we see no need for additional hospital precautions towards LA-MRSA in Denmark at the moment. We think that our data might reduce potential stigmatization of hospital workers with contact to LA-MRSA positive farms at their work places and in their communities.

## Additional files

Additional file 1: Airborne bacteria by active samplers. Concentration of airborne bacteria in different areas at the hospital sampled using different active samplers^a^ (PDF 28 kb)

Additional file 2: Airborne bacteria by passive samplers. Concentration of airborne bacteria in eight different hospital areas, sampled passively with Electrostatic Dust Collectors. (PDF 10 kb)

## Abbreviations

ACI: Andersen Cascade Impactor
CC398: clonal complex 398
EDC: electrostatic dust collectors
GSP: Gesamtstaubprobenahme samplers
LA-MRSA: livestock-associated methicillin-resistant *Staphylococcus aureus*
MALDI-TOF: Matrix-assisted laser desorption-ionization time-of-flight
MRSA plates: MRSA–selective agar plates
MSSA: methicillin sensitive *Staphylococcus aureus*
NA plates: Nutrient agar plates
SA plates: SaSelect agar plates
SSI: Statens Serum Institut
