# Supplementary material for: No apparent transmission of livestock-associated methicillin-resistant Staphylococcus aureus CC398 in a survey of staff at a regional Danish hospital
Source: Antimicrob Resist Infect Control. 2017 Dec 13;6:126. doi: 10.1186/s13756-017-0284-y (PMC5729513; doi:10.1186/s13756-017-0284-y)
Supplement: Supplementary file 1 — Airborne bacteria by active samplers. Concentration of airborne bacteria in different areas at the hospital sampled using different active samplersa (PDF 28 kb) [file 13756_2017_284_MOESM1_ESM.pdf]

ADDITIONAL FILE 1

Concentration of airborne bacteria in different areas at the hospital sampled using different active samplers[a].

**Sampling area**	**Concentration on SA (CFU/m^3) Av (range)**	**Concentration on NA (CFU/m^3) Av (range)**	***n*[a]**	***Staphylococcus* species**	**Other species**	**Number of employees/visitors[b]**
Locker room for female employees	61 (BD-113)	188 (137-239)	3	-	*Micrococcus luteus*	<5/none
	69 (BD-264)	469 (BD-1399)	6	*S. caprae*<br>*S. epidermidis*	*Micrococcus luteus*	None/none
	222 (BD-438)	1061 (BD-2564)	3	*S. epidermidis*<br>*S. hominis*<br>*S. pettenkoferi*	*Kytococcus sedentarius*	<10/none
Patient reception/waiting room	17 (BD-21)	550 (BD-1299)	4	-	*Bacillus infantis*	<10/none
	25 (BD-44)	171 (125-218)	2	-	*Micrococcus luteus*	<5/<5<br>One patient with known MRSA
	26 (BD-44)	BD	2	-	*Micrococcus luteus*	None/none
	9 (BD-22)	BD	2	*S. epidermidis*	*Bacillus sp.*	None/none
	444 (343-545)	308 (BD-533)	2	-	*Corynebacterium stationis*<br>*Micrococcus luteus*	<5/<10
	379 (164-595)	371 (BD-742)	4	-	*Bacillus licheniformis*<br>*Micrococcus luteus*<br>*Microbacterium testaceum*	<5/<10
	30 (BD-50)	124 (BD-166)	2	*S. epidermidis*	*Micrococcus luteus*	<5/<10
	BD	147 (BD-297)	4	*S. epidermidis*		<5/<5
	110 (BD-210)	131 (BD-179)	2	*S. epidermidis*<br>*S. hominis*	*Micrococcus luteus*	<5/<5
Bed washes	78 (BD-285)	159 (BD-416)	6	*S. hominis*	-	<5/none
Cellar with bed and laundry transport	BD	66 (BD-107)	3	-	*Arthrobacter sp.*<br>*Bacillus licheniformis*	<5/<5
Main entrance	Ns	339	1	*S. epidermidis*	*Kytococcus sedentarius*	>20 people
Lunchroom for employees	Ns	643	1	*S. epidermidis*<br>*S. cohnii*<br>*S. hominis*<br>*S. hyicus*<br>*S. pasteuri*<br>*S. pettenkoferi*<br>*S. simulans*<br>*S. warneri*	*Bacillus licheniformis*<br>*Bacillus altitudinis*<br>*Bacillus pumilus*<br>*Kocuria marina*<br>*Kocuria rosea*<br>*Kytococcus sedentarius*<br>*Microbacterium* sp<br>*Micrococcus luteus*<br>*Moraxella osloensis*<br>*Pseudomonas stutzeri*<br>*Solibacillus silvestris*	>20/none
Lunch desk for visitors	Ns	714	1	-	*Bacillus pumilus*<br>*Kytococcus sedentarius*<br>*Microbacterium sp.*	<10 people
Average, *Median*	106, *46*	329, *188*	-	-	-	
Outdoor reference	26 (BD-44)	BD	2	-	-	<10 people

[a]n= number of samples, samples were taken using GSP-sampler, IOM-sampler, BioSampler and ACI-6-stage sampler; [b]number of people present in the sampling area in addition to the occupational hygienist was categorized as: none, less than 5, less than 10, and more than 20. The number of visitors

including patients; SA= SaSelect agar plates; NA=nutrient agar plates; BD=below detection level; ns=not studied.
