# Supplementary material for: No apparent transmission of livestock-associated methicillin-resistant Staphylococcus aureus CC398 in a survey of staff at a regional Danish hospital
Source: Antimicrob Resist Infect Control. 2017 Dec 13;6:126. doi: 10.1186/s13756-017-0284-y (PMC5729513; doi:10.1186/s13756-017-0284-y)
Supplement: Supplementary file 2 — Airborne bacteria by passive samplers. Concentration of airborne bacteria in eight different hospital areas, sampled passively with Electrostatic Dust Collectors. (PDF 10 kb) [file 13756_2017_284_MOESM2_ESM.pdf]

ADDITIONAL FILE 2

Concentration of airborne bacteria in eight different hospital areas, sampled passively with Electrostatic Dust Collectors.

Area	Concentration as measured on SA (CFU/m^2/day)	*Staphylococcus* species
Locker room for female employees	68<br>137<br>376	*S. hominis*<br>*S. epidermidis*<br>*S. xylosus*
Locker room for male employees	1299	*S. hominis*<br>*S. warneri*
Bed washes	68	*S. epidermidis*
Cellar with bed and laundry transport	513	*S. capitis*<br>*S. warneri*
Patient reception/waiting area	34	*S. epidermidis*
Consulting room	155	*S. hominis*<br>*S. warneri*
Average, *Median*	331, *146*	-

SA= SaSelect agar plates.
